# Effects of an aqueous extract of *Eucommia* on articular cartilage in a rat model of osteoarthritis of the knee

**DOI:** 10.3892/etm.2013.1223

**Published:** 2013-07-15

**Authors:** HAI LU, JIANMING JIANG, GUOPING XIE, WENGANG LIU, GUANGBIN YAN

**Affiliations:** 1 Department of Orthopedics, Nanfang Hospital, Southern Medical University, Guangzhou, Guangdong 510515, P.R. China; 2 Guangdong Second Provincial Traditional Chinese Medicine Hospital, Guangzhou, Guangdong 510095, P.R. China; 3 First Affiliated Hospital, Guangzhou Medical College, Guangzhou, Guangdong 510120, P.R. China

**Keywords:** knee osteoarthritis, articular cartilage, *Eucommia*, matrix metalloproteinase-1, matrix metalloproteinase-3, matrix metalloproteinase-13

## Abstract

Osteoarthritis is a common chronic and progressively degenerative joint condition. The stem bark of *Eucommia ulmoides* Oliver (a member of the Eucommiaceae family), which is also known as Du-Zhong, is a traditional Chinese medicine commonly used for the treatment of rheumatoid arthritis. However, the mechanisms underlying the effects of *Eucommia* in the treatment of arthritis of the knee require further study. The present study investigated the effects of an aqueous extract of *Eucommia* on the articular cartilage (by Mankin’s grade) and the levels of matrix metalloproteinase-1 (MMP-1), MMP-3 and MMP-13 in the serum and synovial fluid in a rat model of osteoarthritis. The serum levels of MMP-1, -3 and -13 were measured by double-antibody sandwich enzyme-linked immunosorbent assay (ELISA) at weeks 1, 2 and 4. The levels of MMP-1, -3 and 13 were significantly decreased in the rats treated with *Eucommia* compared with those in the control rats (P<0.05). Histopathological examination results indicated a lower Mankin’s grade in the *Eucommia* group compared with that of the control rats. Therefore, *Eucommia* was demonstrated to have a cartilage-protecting effect in rats with osteoarthritis, potentially by improving cartilage metabolism, regulating the degradation of the extracellular matrix of the articular cartilage, and inhibiting apoptosis in chondrocytes, thereby slowing down joint degeneration.

## Introduction

Knee osteoarthritis (KOA) is a degenerative disease that results in joint pain, stiffness and a reduction in knee function (1). Regardless of continued physiotherapy and the wide use of non-steroidal anti-inflammatory drugs, the disease is of concern, as it affects the mobility of individuals and quality of life. Degeneration and loss of articular cartilage are characteristic features of osteoarthritis. The appearance of fibrillations, matrix depletion, cell clusters and changes in matrix composition reflect the aberrant behavior of resident chondrocytes (2). It is generally believed that degeneration of cartilage in osteoarthritis is characterized by two phases: a biosynthetic phase, during which the cells resident in cartilage, the chondrocytes, attempt to repair the damaged extracellular matrix; and a degradation phase, in which the activity of enzymes produced by the chondrocytes digests the matrix, matrix synthesis is inhibited, and the consequent erosion of the cartilage is accelerated (3,4,5,6). The matrix metalloproteinases (MMPs) are considered important for the chondrolytic processes that contribute to the degenerative changes in osteoarthritis cartilage (7,8). Currently, there is increasing interest in non-synthetic natural drugs that are derived from plant or herbal sources, due to the greater tolerance to such agents and the reduced levels of adverse drug reactions (9). *Eucommia ulmoides* Oliver is a native Chinese medicinal herb, the bark of which has long been utilized for the treatment of arthritis in China. However, the mechanisms of action of *Eucommia* remain unclear. In the present study, the effects of an aqueous extract of *Eucommia* on the articular cartilage were investigated in a rat model of KOA. Mankin’s grade was evaluated and the serum and synovial fluid levels of MMP-1, -3 and -13 were measured.

## Materials and methods

### Medicinal material

*Eucommia* bark (500 g; origin, Sichuan, China) was purchased from Zhixin Chinese Herbal Co., Ltd. (batch no. 120701; Guangzhou, China). The aqueous extract of *Eucommia* was prepared as described previously (10). *Eucommia* bark (500 g) was soaked in distilled water for 30 min, then heated to boiling for 10 min, simmered for 30 min and the dregs were filtered. The procedure was repeated twice, all decoction was collected which yielded a final concentration of ~0.5 g crude extract/ml (1,000 ml).

### Instruments

An inverted microscope (BX51TRF; Olympus Optical Co., Ltd., Tokyo, Japan), a high-speed centrifuge (3k30; Sigma, St. Louis, MO, USA), a microplate reader (MK352, Hercules, CA, USA), a microtome (Leica RM2235; Leica Biosystems, Wetzlar, Germany) and a Leica TP1020 Auto Processor System (Leica Biosystems) were used in the study.

### Animals and KOA model

A total of 54 Sprague-Dawley rats (weight, 180–220 g) comprising 27 males and 27 females, were obtained from the Laboratory Animal Center, Guangzhou University of Traditional Chinese Medicine [license no. scxk(Yue)2008–0020; Guangzhou, China]. Rats were housed in a humidity-controlled room at 20°C with access to fresh water and standard laboratory food *ad libitum*. All experimental procedures were approved by the Animal Care and Use Committee, Guangzhou University of Traditional Chinese Medicine (2008C067). Osteoarthritis (OA) was induced in rats by section of the anterior cruciate ligament of the right knee through a stab incision (11), KOA was not induced in the blank group.

### Experimental procedures

The rats were randomly divided into three groups: the blank, *Eucommia* and control groups. In the blank group, 18 normal rats were fed *ad libitum*; whereas in the *Eucommia* group, an aqueous extract of *Eucommia (*6 ml/kg/day) was administered to each rat (n=22) for 4 weeks. The *Eucommia* dosage was based on the surface area of the rats, which was calculated by the Meeh-Rubner formula (12). In the control group, distilled water (6 ml/kg/day) was administered to each rat (n=22) for 4 weeks (Table I).

### Matrix metalloproteinase-1 (MMP-1), MMP-3 and MMP-13 levels

Six rats were randomly selected from each group 1, 2 and 4 weeks following the initiation of treatment. Blood was sampled from the retro-orbital plexus of the selected rats. In order to obtain the synovial fluid, the right knee of each rat was cut and the joint cavity was exposed under aseptic conditions. The cavity was then lavaged with 1 ml saline and 0.5 ml synovial fluid was aspirated. The synovial fluid specimens were centrifuged at 4,500 r/min for 10 min, then the supernatant was stored in Eppendorf tubes at −80°C. The serum and synovial fluid levels of MMP-1, -3 and -13 were measured by double-antibody sandwich enzyme-linked immunosorbent assay (ELISA).

### Histopathological findings

Samples of articular cartilage were obtained from the lateral tibial condyle of each rat and histopathologically graded (Table II) (13).

### Statistical analysis

Statistical analysis was performed using SPSS software, version 13.0 (SPSS, Inc., Chicago, IL, USA). Quantitative variables are expressed as the mean ± standard deviation. One-way analysis of variance, the Student’s t-test and correlation analyses were used, and the differences were determined by the t-test and the least significant difference (LSD). P<0.05 was considered to indicate a statistically significant difference.

## Results

### Macroscopic evaluation

In the blank group, joint swelling was not identified and the articular cartilage of the knee appeared smooth, lustrous, pale blue and translucent. However, in the control group, examination indicated swelling of the right knee joint and congestion. Furthermore, the synovial fluid was opaque and pale yellow, the cartilage surface was dull and rough, significant ulcers and fissures were observed, and the gross exposure of subchondral bone and ulcerations was occasionally demonstrated. In addition, congestion, hypertrophy and edema of the synovial membrane were identified in this group. In the *Eucommia* group, the knee articular cartilage was pale and close to normal in appearance; however, the cartilage was softer compared with that of the control group and an occasional crack was identified in the surface. Moreover, mild hyperplasia of the synovial lining was identified in the *Eucommia* group.

### Histopathology

The articular cartilage samples from the rats in each group were evaluated using Mankin’s grades (Table III) (13).

### Morphological changes in the articular cartilage after 4 weeks of treatment

**Figure 1:**
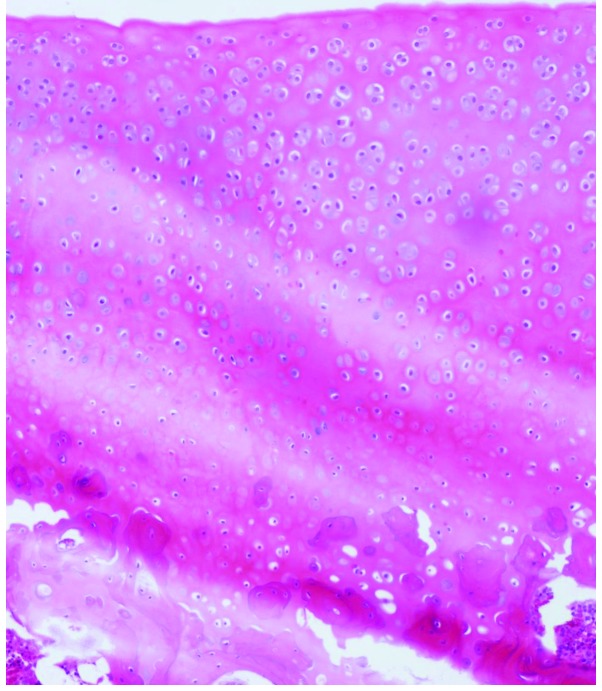
Morphological changes in the articular cartilage after 4 weeks of treatment in the control group.

**Figure 2:**
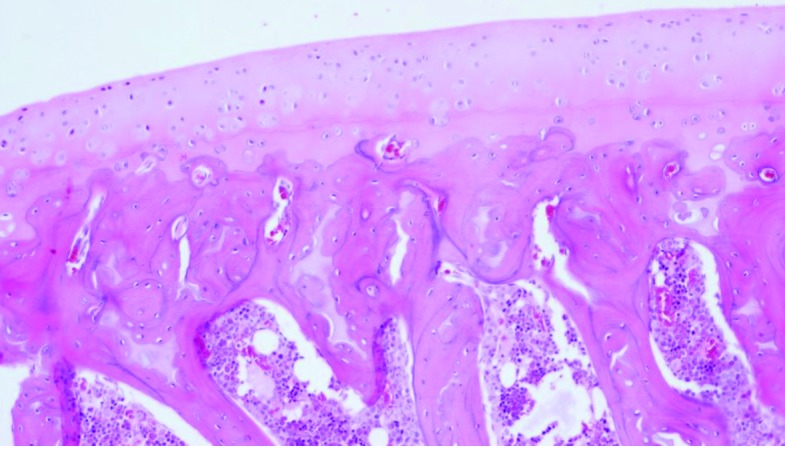
Morphological changes in the articular cartilage after 4 weeks in the blank group.

**Figure 3:**
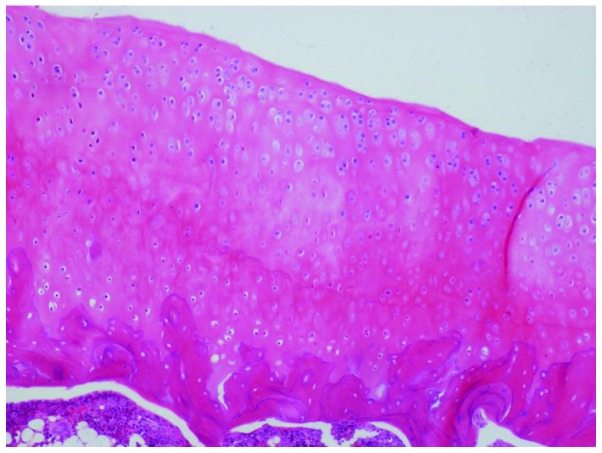
Morphological changes in the articular cartilage after 4 weeks of treatment in the *Eucommia* group.

In the control group, the surface of the articular cartilage was rough and indications of erosion and exfoliation, and visible cracks across the surface, were observed. Chondrocyte cluster formation was identified and the tide line was not continuous. In addition, stromal staining appeared uneven, decreased and was absent in intensity. Fibrous tissue proliferation and clusers were observed (Fig. 1). In the blank group, the surface of the cartilage was smooth and the cell layers were clearly defined. In addition, the cells were uniform and arranged neatly. Staining of the stroma appeared even (Fig. 2). In the *Eucommia* group, the articular cartilage had structural integrity. The staining of the stroma was occasionally uneven. Furthermore, there was a decreased number of chondrocytes, and mild to moderate damage of the articular cartilage was evident (Fig. 3).

### Levels of MMP-1, -3 and -13

In the synovial fluid of the *Eucommia* group, the levels of MMP-1, -3 and -13 were significantly lower at 4 weeks compared with those of the control group (P<0.05; Table IV). In addition, the serum levels of MMP-1, -3, and-13 in the *Eucommia* group were significantly lower than those of the control group (P<0.05; Table V).

## Discussion

Approximately 10% of individuals >55 years of age are affected by osteoarthritis in the UK and the Netherlands, one-quarter of whom are severely disabled (14). The condition is characterized by degeneration of the articular cartilage and subsequent changes to the subchondral bone. The underlying mechanisms remain unknown, but the glycosaminoglycan-proteoglycan matrix is proposed to be important in the progression of the disease (15). X-ray examinations of osteoarthritis do not indicate early cartilage abnormalities. However, the detection of potential biomarkers in the synovial fluid may be a precise method that would facilitate the early diagnosis of the disease.

Among the various biological markers associated with OA, MMPs are important in cartilage degradation in human joint diseases, and they function downstream of the OA signaling pathways (16,17). Following excretion from the cell as inactive pro-forms, MMPs are converted into the active enzymes, which are inhibited by the reversible binding of MMPs with specific inhibitors, including tissue inhibitors of metalloproteinases (TIMPs) (18). The activation of MMPs is closely associated with cartilage degradation. One or more MMPs may digest the majority of the matrix components *in vitro*(19). In addition, elevated levels of MMPs are identified in OA cartilage at the site of cartilage destruction, and specific digested parts of MMPs may be identified in synovial fluid samples taken from patients with OA (20). MMP-2 and -9 are particularly important in cartilage degradation as they degrade a variety of collagens, including basement membrane type V collagen and denatured fibrillar type I collagen (21,22). Therefore, MMPs may potentially be utilized as a promising biological markers of OA.

The stem bark of *Eucommia ulmoides* Oliver (a member of the Eucommiaceae family), which is also known as Du-Zhong, is commonly utilized in traditional Chinese medicine for the treatment of rheumatoid arthritis (23,24). Furthermore, studies have shown that crude flavonoids and polysaccharides from *Eucommia* extracts are the major components that contribute to the anti-bacterial, -inflammatory, -oxidation, -aging and -cancer activities, among numerous other physiological functions, of the extracts (25,26).

Previous studies have suggested that *Eucommia,* combined with various plant-derived medicines, is effective in decreasing the rate of apoptosis in chondrocytes (27). However, the effect of *Eucommia* treatment on articular cartilage degeneration remains unclear. The present study observed the effects of an aqueous extract of *Eucommia* on the articular cartilage in a rat model of KOA. The histopathology and MMP-1, -3 and -13 levels in the serum and synovial fluid were investigated to identify the possible involvement of *Eucommia* in the protection of articular cartilage.

OA was surgically induced in the knee joint as described previously (11). One week following surgery, the control group demonstrated joint capsule adhesions and the cartilage did not exhibit a glossy surface, but was smooth and yellow. Two weeks following surgery, the cartilage was yellow, but the color had darkened and fissures of varying size were identified. Four weeks following surgery, the structural damage of the cartilage was severe, and the femoral external condyle and tibial plateau edge markedly demonstrated hyperplasia. These results were consistent with the pathomorphological changes that occur in osteoarthritis; therefore, in the present study, the OA model was successfully established. According to Mankin’s criteria (28), the grading of cartilage in the control group was significantly higher compared with that of the *Eucommia* group (P<0.05).

There is only a small volume of synovial fluid in the knee joints of rats, which is difficult to extract. Therefore, in the present study, saline was injected into the rat joint cavity. The joint irrigation fluid was then extracted and the levels of MMPs in the fluid were measured. The results of the present study demonstrated that the aqueous *Eucommia* extract significantly reduced the levels of MMP-1, -3 and -13 in the joint irrigation fluid and in the blood of the rats. Therefore, *Eucommia* may be important in the inhibition of inflammatory factors and in preventing the degradation of the cartilage matrix in rats with OA.

An increase in the number of studies demonstrating the effect of *Eucommia* in the progression of OA has resulted in an interest in drugs that affect bone metabolism, and drugs that may slow down or even halt the process of joint degeneration. However, the degradation of the cartilage extracellular matrix that is observed in OA is a complex process. The present study explored the effects of *Eucommia* on the levels of MMP-1, -3 and -13 in rats with osteoarthritis; however, the underlying mechanisms remain unclear.

## Figures and Tables

**Table I:** Rat groups and administration.

Groups	No. of rats	Treatment	Methods	Dosage (ml/kg/day)
Blank	18	-	-	-
Control	18	Distilled water	Gavage	6
*Eucommia*	18	*Eucommia* decoction	Gavage	6

**Table II:** Histological and histochemical grading.

Variable	Grade
Structure	
Normal	0
Surface irregularities	1
Pannus and surface irregularities	2
Clefts to transitorial zone	3
Clefts to radial zone	4
Clefts to calcified zone	5
Complete disorganization	6
Cells	
Normal	0
Diffuse hypercellularity	1
Cloning	2
Hypocellularity	3
Safranin-0 staining	
Normal	0
Slight reduction	1
Moderate reduction	2
Severe reduction	3
No dye noted	4

**Table III:** Morphological changes in the articular cartilage.

Groups	No. of rats	Mankin’s grade
Blank	18	0.25±0.21
Control	18	4.87±0.83^a^
*Eucommia*	18	2.64±0.07^a^

a P<0.05, compared with the control group.

**Table IV:** Levels of MMP-1, -3 and-13 in the synovial fluid at various time points.

Groups	No. of rats	Time (weeks)	MMP-1	MMP-3	MMP-13
Blank	18	-	-	-	-
Control	18	1	3.79±0.18	3.17±0.44	0.39±0.11
		2	5.01±0.93	4.07±0.11	0.55±0.84
		4	5.89±0.65	5.12±0.34	0.67±0.21
*Eucommia*	18	1	3.76±0.02	3.16±0.65	0.38±0.47
		2	3.76±0.77	4.01±0.29	0.31±0.61
		4	2.81±0.93^a^	2.46±0.75^a^	0.24±0.81^a^

a P<0.05, compared with the control group.

MMP, matrix metalloproteinase.

**Table V:** Levels of MMP-1, -3 and -13 in the serum following surgery at various time points.

Groups	No. of rats	Time (weeks)	MMP-1	MMP-3	MMP-13
Blank	18	-	-	-	-
Control	18	1	10.95±0.66	11.11±0.29	1.01±0.12
		2	11.38±0.79	12.26±0.33	1.56±0.74
		4	13.56±0.17	12.38±0.89	1.96±0.22
*Eucommia*	18	1	8.26±0.12	10.22±0.31	0.89±0.46
		2	6.10±0.54	7.18±0.82	0.76±0.33
		4	5.16±0.42^a^	5.01±0.27^a^	0.41±0.77^a^

a P<0.05, compared with the control group.

MMP, matrix metalloproteinase.
